# A simple, step-by-step guide to interpreting decision curve analysis

**DOI:** 10.1186/s41512-019-0064-7

**Published:** 2019-10-04

**Authors:** Andrew J. Vickers, Ben van Calster, Ewout W. Steyerberg

**Affiliations:** 1 Department of Epidemiology and Biostatistics, Memorial Sloan Kettering Cancer Center, 485 Lexington Avenue, 2nd Floor, New York, NY 10017 USA; 2 Department of Development and Regeneration, KU Leuven, Oude Markt 13, 3000 Leuven, Belgium; 3 Department of Biomedical Data Sciences, Leiden University Medical Center, Albinusdreef 2, 2333 ZA Leiden, Netherlands

**Keywords:** Net benefit, Decision curve analysis, Educational paper

## Abstract

**Background:**

Decision curve analysis is a method to evaluate prediction models and diagnostic tests that was introduced in a 2006 publication. Decision curves are now commonly reported in the literature, but there remains widespread misunderstanding of and confusion about what they mean.

**Summary of commentary:**

In this paper, we present a didactic, step-by-step introduction to interpreting a decision curve analysis and answer some common questions about the method. We argue that many of the difficulties with interpreting decision curves can be solved by relabeling the *y*-axis as “benefit” and the *x*-axis as “preference.” A model or test can be recommended for clinical use if it has the highest level of benefit across a range of clinically reasonable preferences.

**Conclusion:**

Decision curves are readily interpretable if readers and authors follow a few simple guidelines.

## Introduction

Decision curve analysis is a method to evaluate prediction models and diagnostic tests that was introduced by Vickers and Elkin in a 2006 publication in *Medical Decision Making* [1]. The method sought to overcome the limitations of both traditional statistical metrics, such as discrimination and calibration, which are not directly informative as to clinical value, and full decision analytic approaches, which are too unwieldy to be used in regular biostatistical practice.

In brief, decision curve analysis calculates a clinical “net benefit” for one or more prediction models or diagnostic tests in comparison to default strategies of treating all or no patients. Net benefit is calculated across a range of threshold probabilities, defined as the minimum probability of disease at which further intervention would be warranted, as net benefit = sensitivity × prevalence – (1 – specificity) × (1 – prevalence) × *w* where *w* is the odds at the threshold probability. For a prediction model that gives predicted probability of disease *p̂*, sensitivity and specificity at a given threshold probability *p*_t_ is calculated by defining test positive as *p̂ ≥ p*_t_*.* Net benefit differs from accuracy metrics such as discrimination and calibration because it incorporates the consequences of the decisions made on the basis of a model or test. For more on the background to decision curve analysis, see Vickers et al. [2].

Recent years have seen an explosion of interest in and practical use of decision curve analysis. The paper has been widely cited, with > 1000 citations on Google Scholar as of May 2019. Decision curve analysis has been recommended by editorials in many top journals, including *JAMA*, *BMJ*, *Annals of Internal Medicine*, *Journal of Clinical Oncology*, and *PLoS Medicine* [2–6].

That said, there does appear to be widespread misunderstanding of and confusion about decision curve analysis. For instance, a well-respected epidemiologist claimed that he had yet to find more than a couple of people in the world who could explain what decision curves meant and that he himself was not clear on their interpretation. We have also attended meetings where presenters have shown a decision curve slide and then commented that they themselves did not actually understand it.

Here, we present a didactic, step-by-step introduction to interpreting a decision curve analysis. Each step aims to give increasing understanding. Mastery of any step will give at least some insight into a published decision curve, although understanding all steps will naturally provide the greatest insight. In contrast to prior editorials, which are aimed predominately at investigators wishing to report a decision curve analysis, our main audience here is readers who wish to understand a published decision curve. In this paper, we ask readers to consider individual patient scenarios, such as a patient who has young children and is worried about cancer. Note that such examples are for didactic purposes only: as pointed out below, decision curves are a research tool and are not for direct use in the clinic. Note also that we will not comment further on how to calculate decision curves, nor comment on their mathematical properties: readers are referred to the appropriate methodological literature [1, 7, 8] and to www.decisioncurveanalysis.org

We will use as our main example a study of prostate cancer biopsy, a topic that has been subject to several papers reporting decision curves, with a multi-institutional comparison of two prediction models being but one example [9]. As background, men undergoing screening with prostate-specific antigen (PSA) are generally advised to have a biopsy if their PSA is elevated, for instance, a value of 3 ng/mL or higher. However, only a small proportion of such men have high-grade cancer, the kind that benefits from early treatment. In contrast, low-grade cancer is considered to constitute overdiagnosis, and, of course, no urologist would recommend a biopsy of a man without cancer. Researchers have tried to find additional markers that could predict high-grade cancer in men with elevated PSA. The idea is that any man with an elevated PSA would undergo a second test, and only be referred to biopsy if that indicated a high risk of aggressive disease. In our hypothetical study, the prevalence of high-grade cancer is 10%. We suppose that the study evaluated both a binary diagnostic test (sensitivity 40%, specificity 90%) and a statistical prediction model based on several markers that give an output in terms of a predicted probability of disease and has an area under the curve (AUC) of 0.79. We calculate the decision curves following the methods first described in the Vickers and Elkin paper [1]. We then address some frequently asked questions about decision curves.

## Interpreting a decision curve analysis

### Step 1: Benefit is good

**Fig. 1:**
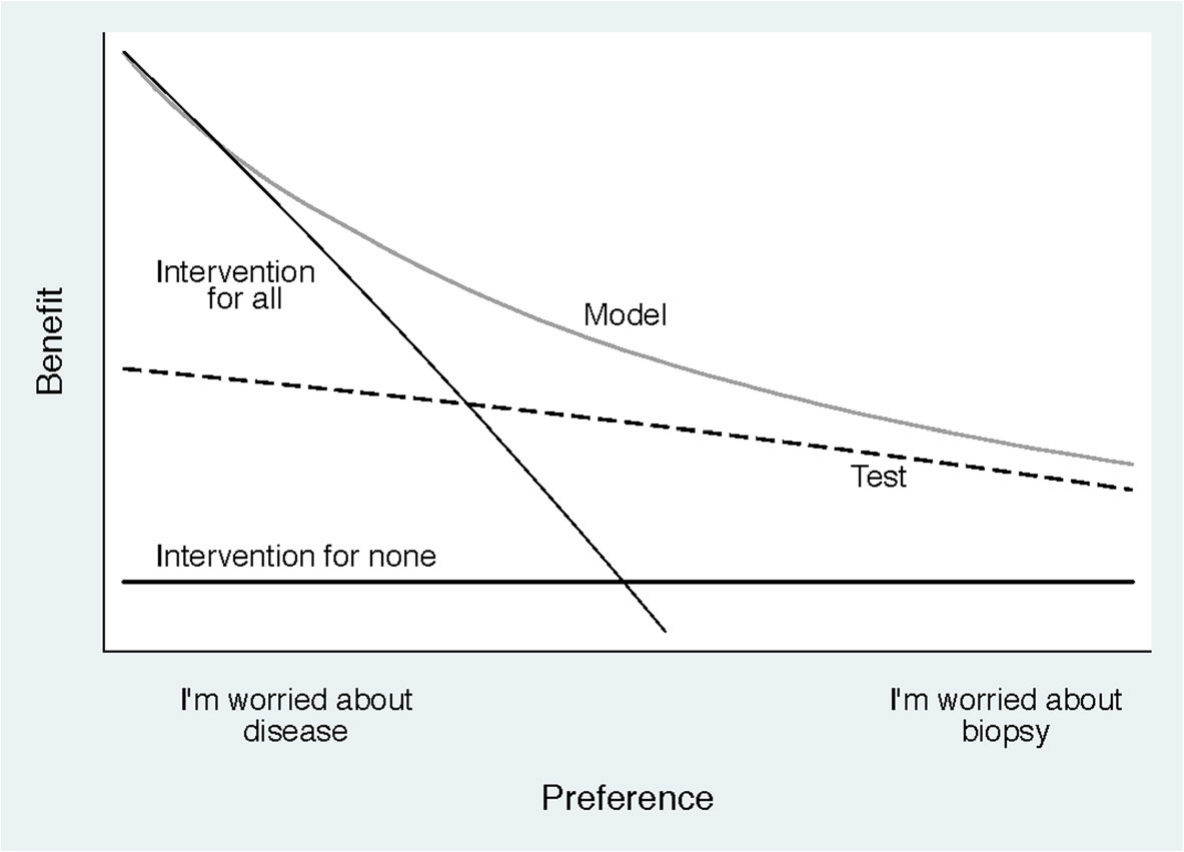
A decision curve plotting benefit against preference

Figure 1 shows only the most essential elements of a decision curve analysis. The result for the prediction model is the light gray line, and the diagnostic test is the dashed line. The two other lines are for “intervention for all” (thin black line) and “intervention for none” (thick black line).

“Intervention” is used in a general sense: it might refer to drugs or surgery, but it could also encompass lifestyle advice, additional diagnostic workup, or subsequent monitoring. Indeed, intervention reflects any action that a patient at high risk from a model, or getting a positive result on a diagnostic test, would consider to improve their health, or their life in general. The exact intervention depends on the clinical setting. In our study of prostate cancer in men with elevated PSA, intervention would mean prostate biopsy. To give other examples, in a study of infection, intervention might be giving antibiotics; in a study of heart disease prevention, intervention might be giving statins. In a study of palliative surgery for advanced cancer, with an endpoint of death within 3 months; however, the idea would be to avoid surgery in patients at high risk and intervention would be “best supportive care.” Note that in the original paper, describing decision curve analysis, and in many empirical applications, the word “treat” is used in place of intervention.

Decision curve analysis includes results for “intervention for all” and “intervention for none” because these are often reasonable clinical strategies [10, 11]. To give a specific example, one reasonable strategy in the prostate biopsy study would be to biopsy all patients with elevated PSA irrespective of the results of the diagnostic test or prediction model. Indeed, this is generally what happens in contemporary practice, where men who have a PSA above a certain threshold are routinely biopsied without additional testing. On the other hand, we might imagine a study of men with low PSA, who are not subject to biopsy in routine clinical practice. Some of these men do have high-grade prostate cancer, and researchers might be investigating a suitable test. In this case, the reference strategy would be “intervention for none.”

On the figure, the *y*-axis is benefit and the *x*-axis is preference. The benefit of a test or model is that it correctly identifies which patients do and do not have disease (in our example, high-grade cancer). Preference refers to how doctors value different outcomes for a given patient, a decision that is often influenced by a discussion between the doctor and that patient. Both preference and benefit are described in further detail below: at this stage, it is only important to know that benefit is good and that preferences vary. It is easily seen that the light gray line, corresponding to the prediction model, has the highest benefit across a wide range of values of preference. Hence, we can conclude that, except for a small range of low preferences, intervening on (i.e., biopsying) patients on the basis of the prediction model leads to higher benefit than the alternative strategies of biopsying all patients, biopsying no patients, or only biopsy those patients who are positive on the diagnostic test. For the prostate biopsy study, the conclusion is that using the model to determine whether patients should have a biopsy would lead to improved clinical outcome.

### Step 2: Preference refers to how doctors value different outcomes for their patients

Following a consultation and a discussion with some patients, a doctor might be particularly worried about missing disease; for other patients, the doctor may be more concerned about avoiding unnecessary intervention. Doctors may also vary in their propensity to intervene, some being more conservative, others more aggressive. In Fig. 1, the extremes of the *x*-axis for preference are “I’m worried about disease” and “I’m worried about biopsy.” In the case of prostate cancer biopsy, a doctor who, for a given patient, has a preference towards the left end of the *x*-axis weighs the relative harm of missing a high-grade cancer as much greater than the harm of unnecessary biopsy. This may be, for instance, because the patient is younger and has school-age children, and so very much prioritizes finding any lethal cancer at a curable stage: this patient is clearly “worried about disease,” consistent with a low threshold for continuing diagnostic workup. A doctor with a preference for a given patient towards the right of the *x*-axis wants to avoid biopsy if possible. This might reflect a patient who does not like the idea of invasive medical procedures or a doctor treating an older patient and who is skeptical about the value of early detection in that population: they are “worried about biopsy” and will opt for biopsy only if the patient is at particularly high risk.

This helps us take our interpretation a little bit further. We can see that the model has higher benefit than the other approaches, apart from doctors who fall in the “very worried” category, for whom the benefit is actually slightly higher for the strategy of “intervention for all.” This makes intuitive sense: a patient with an elevated PSA who has a strong preference for early identification of potentially lethal cancer might want to go straight ahead and get a biopsy rather than depend on a second model or test that is not 100% accurate.

### Step 3: The unit of preference is threshold probability

Our model gives a patient’s predicted probability of high-grade cancer. One might assume that if the model estimated the patient’s risk as 1%, both the patient and the doctor would agree that there was no need for biopsy; if the risk was 99%, however, the doctor would advise and the patient accept that biopsy was indicated. Comparable conclusions would be drawn if the risks were 2% versus 98%. We might imagine that we vary the risks, counting up from 2% and down from 98% until the doctor is no longer sure. For instance, a doctor might say “Thinking about this patient, I wouldn’t do more than 10 biopsies to find one high-grade cancer in patients with similar health and who think about the risks and benefits of biopsy vs. finding cancer in the same way. So if a patient’s risk was above 10% I do a biopsy, otherwise, I just carefully monitor the patient and perhaps do a biopsy later if I saw a reason to.”

**Fig. 2:**
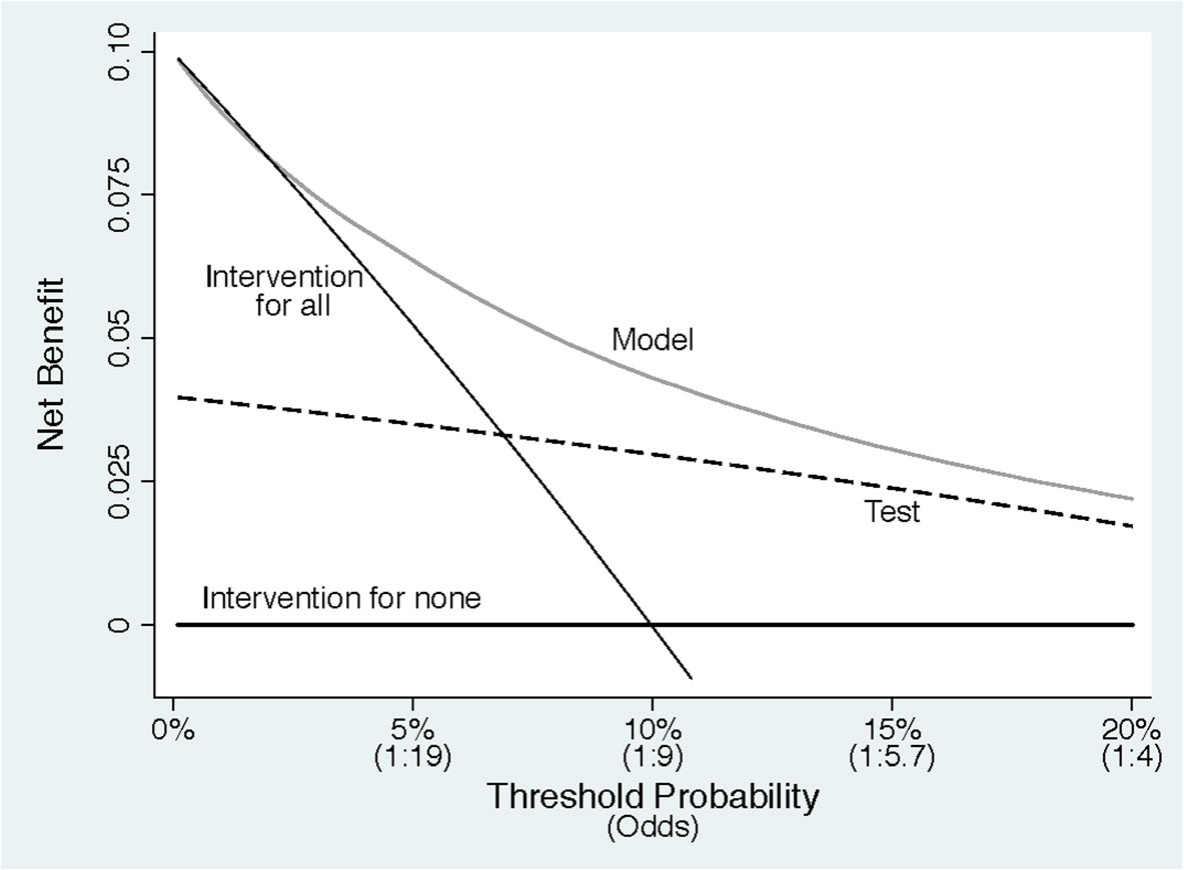
A decision curve plotting net benefit against threshold probability

The relationship between preference and threshold probability is perhaps the easiest to see when using the odds. The risk of 10% is an odds of 1:9, so in using a threshold probability of 10%, the doctor is telling us “missing a high-grade cancer is 9 times worse than doing an unnecessary biopsy” [2]. This can be interpreted as the “number-needed-to-test,” that is, 10% is a number-needed-to-test of 10. Figure 2 shows threshold probabilities on the *x*-axis. Odds are also shown for didactic purposes, although these are omitted when presenting decision curves. This helps us to understand our previous conclusion that patients who are particularly worried about disease do not benefit from using the model. We can now see that it is only if threshold probabilities are less than 2 or 3% that we should avoid using the model. That would be a stretch in prostate cancer, where biopsy is invasive, painful, and associated with the risk of sepsis. However, such a low threshold might be plausible in some other scenarios, for instance, biopsy for skin cancer, which is a far less risky and less invasive procedure. Note also that the curve is only plotted up to 20%. This is because, given the relative risks of missing a high-grade prostate cancer compared to the harms of biopsy, we would consider it unreasonable for any patient or doctor to demand greater than 20% risk before accepting biopsy. The plausible range of thresholds hence depends critically on context. Elsewhere, we describe in detail the process by which a reasonable range of thresholds can be agreed upon [2].

### Step 4: Benefit is actually net benefit

Figure 2 also shows the correct units for benefit, what is known as “net benefit.” The “net” in “net benefit” is the same as in “net profit,” that is, income minus expenditure. If, say, a wine importer buys €1m of wine from France and sells it in the USA for $1.5m, then if the exchange rate is €1 to $1.25, the net profit is income in dollars (1.5m) − expenditure in euros (1m) × exchange rate (1.25) = $250,000. Leaving aside, for the sake of simplicity, the issue of risk and the time and trouble to trade, this is equivalent to being given $250,000 without having to do any trading. In the case of diagnosis, the income is true positives (e.g., finding a cancer) and the expenditure is false positives (e.g., unnecessary biopsies), with the “exchange rate” being the number of false positives that are worth one true positive. The exchange rate will depend on the relative seriousness of the intervention and outcome. For instance, we will be willing to conduct more unnecessary biopsies to find one cancer if the biopsy procedure is safe vs. dangerous or the cancer is aggressive vs. more indolent. The exchange rate is calculated, as explained above, from the threshold probability. Another analogy is with net health benefit or net monetary benefit, which both depend on the willingness to pay threshold in their exchange of benefits in terms of health and costs [12].

The unit of net benefit is true positives. A net benefit of 0.07, for instance, means “7 true positives for every 100 patients in the target population.” So just like in the example of net profit for the wine trader, a net benefit of 0.07 would be the equivalent of identifying 7 patients per 100, all of whom had disease. In the prostate biopsy example, a 0.07 net benefit would be equivalent to a strategy where 7 patients per 100 were biopsied and all were found to have high-grade tumors. Also comparable to the business example, where a profit of $250,000 could result from various combinations of income and expenditure, a net benefit of 0.07 could result from different combinations of true and false positives.

### Step 5: Net benefit can also be expressed as interventions avoided

**Fig. 3:**
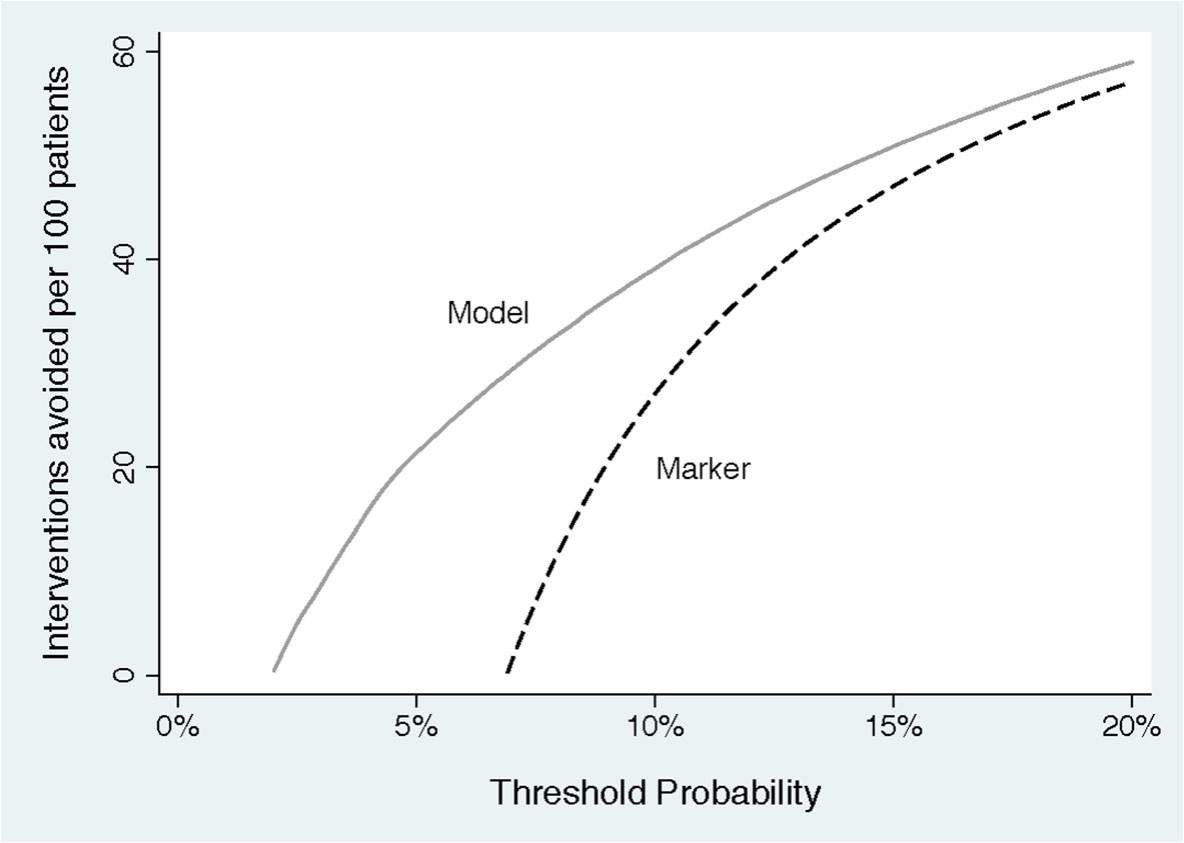
A decision curve plotting decrease in interventions against threshold probability

In many scenarios, the most common strategy is to “intervention for all” rather than to “intervention for none.” Indeed, this is the case for our prostate cancer example, where urologists routinely biopsy all patients with an elevated PSA. In these scenarios, a model or test would aim to reduce unnecessary intervention. Net benefit can be expressed in terms of true negatives rather than true positives. Figure 3 shows an example of this type of decision curve. This could be interpreted that, at a risk threshold of 10%, use of the prediction model would be the equivalent of a strategy that reduced the number of unnecessary biopsies by about 40 per 100 without missing biopsy for any patients with high-grade cancer. Expressing net benefit in terms of avoided unnecessary diagnostic procedures or avoided unnecessary treatments is recommended if the reference strategy is “intervention for all.” Note that doing so does not change any conclusions as to which model or test has the highest net benefit.

## Some common questions about interpreting decision curves

1. *What if we do not know the threshold probability?* A threshold probability is necessary to use any model or test for decision-making. If our prostate cancer prediction model gave a predicted risk of, say, 40%, and no one knew whether that was high or low, and therefore could not tell whether biopsy was indicated, then the model could not be used to make a decision. As a result, the question of using a decision analytic technique such as decision curve analysis to evaluate the model would be redundant.
2. *How is treatment effect taken into account?* In most decision curves, the effect of treatment is implicit and is incorporated into the threshold probability. In general, the more effective the treatment, the lower the threshold probability: larger treatment effects imply lower thresholds. In the prostate cancer example, the diagnosis of high-grade disease would be considered more important, and hence probability thresholds would be lower, if diagnosing and treating high-grade cancer had a larger effect on life expectancy. As another simple example, consider a decision curve to predict heart attack, where patients at high risk are given a prophylactic drug. Imagine that the drug reduced the relative risk of a cardiac event by 10% and was associated with an absolute 1% risk of a serious side-effect such as a stroke or gastrointestinal hemorrhage. If we assume that cardiac events and serious side-effects are equally harmful, then the minimum threshold probability to justify treatment would be 10%. This is because a 10% relative risk reduction from 10% is 1% in absolute terms, so the reduction in the risk of a cardiac event would be the same as the increase in the risk of stroke. However, if the drug were more effective, say a 20% relative risk reduction, then the minimum threshold probability would be 5%. That said, some models predict not absolute risk but treatment benefit, that is, “patient X is predicted to have a 2% absolute reduction in risk” rather than “patient X has a 20% absolute risk of the event.” An alternative version of decision curve analysis is available for such models [13].
3. *How much of a difference in curves is enough?* In classical decision theory, the strategy with the highest expected utility should be chosen, irrespective of the size or statistical significance of the benefit. Theoretically, any improvement in net benefit is therefore worth having. That said, a straightforward decision analysis does not take into account the time and trouble required to obtain data for and implement a model. Now, if a model required a variable from an invasive medical procedure associated with non-trivial risk, we would likely not use the model if it had only a small improvement in net benefit. There are two approaches to this problem. First, as described in the original paper on net benefit [1], investigators can formally incorporate harm associated with the model or test into a decision curve. In brief, the investigators ask: “if the test/model was perfect, how many patients would I subject to it in order to find one true case (e.g., a cancer)?”. The reciprocal of this number is known as “test harm” and is subtracted from net benefit. Alternatively, the investigators can look at differences in net benefit, or interventions avoided, and make an informal judgment; this is related to the concept of “test trade-off” [14]. Using the data shown in Figure 3, one might ask whether it is worth calculating the model for 100 patients in order to prevent 39 biopsies, or whether it is worth using the model rather than the test to prevent 5 biopsies. The answer to those questions depends on the sort of information required for the model and for the test, such as whether an invasive, harmful, or expensive procedure was required.
4. *Should there be confidence intervals or p values for decision curves?* Statistical significance and confidence intervals are not important concepts in classical decision theory. This can be described in brief as follows. A decision-maker should start by considering all reasonable options for a given decision problem. Which options count as “reasonable” might well include consideration of statistical significance. But when choosing between different options, the most rational choice is (in general) that with the highest expected utility, irrespective of statistical significance. As a simple thought experiment, consider an individual who had to rush home for an appointment, could take either one of two bus routes, and happened to have a dataset of the times for each route. If the mean times home were 30 vs. 35 min, with similar distributions and variances, the individual would be advised to take the quicker route home, even if the difference was not statistically significant and the confidence interval for the difference in times overlapped with zero. As a result, few published decision curves incorporate confidence intervals. Confidence intervals may be useful in certain scenarios, for instance, to determine whether more research is required. Methods for the calculation of confidence intervals have been published [7].
5. *How can a model be harmful if area under the curve (AUC) is better than 0.50? If one model has a better AUC than another, how can it have a worse net benefit?* Net benefit takes into account both discrimination (AUC) and calibration [15]. To give a simple example, imagine that we took the predictions from the prostate cancer and divided by 10. Although this would have no effect on AUC—patients with a higher risk are more likely to have high-grade cancer than patients at lower risk—it would have obvious effects on clinical value: we might tell a patient at 40% risk that risk was only 4%. With that risk estimate, he would elect not to have a biopsy, leading to an important risk of missing an aggressive cancer.
6. *Why is “intervention for all” or “intervention for none” a relevant comparison?* Intervening for all or no patients, irrespective of test or model results are reasonable clinical strategies in many scenarios. A test or model must be found superior to both of these strategies to justify being used in clinical practice [10]. There are several examples in the literature demonstrating the value of comparing models to intervention for all or no patients. For instance, Nam et al. [9] found that a prostate biopsy model had a lower net benefit than biopsying all men at elevated risk, because the model underestimated the risk of cancer.
7. *Can a decision curve analysis substitute a traditional decision analysis or cost-effectiveness analysis?* Decision curve analysis is much quicker and easier than a full decision analysis because it requires fewer parameters to be specified (indeed, only one, the reasonable range of threshold probabilities). However, doing so involves simplifying assumptions. If the results of the decision curve analysis are very clear, for instance, that a model has no benefit, this may obviate the need for a more complex decision analysis. On the other hand, if the results are more equivocal, there may be a case for a decision analysis with a more completely specified list of parameters for benefits, harms, and costs.
8. *Can you use a decision curve to choose the best threshold?* This is a frequent and fundamental misunderstanding. Investigators have sometimes written statements such as “the model was superior in the range 30 – 40%; therefore patients should choose intervention if their probability from the model is greater than 30 – 40%.” This reverses the relationship between threshold probability and evaluation of a model. Investigators should first work out a clinically reasonable range of threshold probabilities, based on considering the relative harms of avoid intervention for a patient with disease versus unnecessarily intervening on a patient who is disease free. They should then determine whether the net benefit of their model or test is better than alternatives across this range of threshold probabilities.
9. *How do you use a decision curve analysis in the clinic?* A decision curve analysis has no more direct clinical applicability than, say, the *p* value and overall absolute risk reduction from a trial of a new drug. In the drug trial, a *p* value might be used to conclude “the drug works” and the overall absolute risk reduction to judge that “the benefit of the drug outweighs the harms.” In such a case, a doctor would then give the drug to patients where indicated, without looking up the trial results each time. In a similar way, a decision curve is used to evaluate whether a model or test would be of benefit in the clinic. If results are positive, then the model or test can be used with appropriate patients as part of shared decision-making without any need to refer back to the original decision curve.
10. *Do I need to know the threshold probability for an individual patient before I use the results of a decision curve and use the results of a test or model?* This is not how decision curves are intended to be used. If a model or test has the highest net benefit across the entire range of reasonable threshold probabilities, then clearly that model or test should be used irrespective of patient preference. If the optimal approach depends on the threshold probability, then the typical conclusion would be that the model or test is of unproven benefit or that it is only useful in settings where we assume a specific range of preferences. A more formal decision analysis might involve elicitation of individual preferences from a study sample and integration of utilities across a distribution of these preferences.

## Conclusion

A PubMed search for “decision analysis” restricted to 2017 retrieves 311 papers; a comparable search for “decision curve analysis” retrieves 95. Given that few of decision curve papers would have involved a decision analytic methodology if not for the availability of a straightforward analytic technique, this means that decision curve analysis is responsible for an important increase in the use of decision analysis in the medical literature. A greater understanding of decision curve analysis is therefore not only of inherent value, but will also lead to a greater appreciation of decision analytic principles in the research community as a whole.

When investigators have indicated to us that “decision curve analysis is hard to understand,” it is clear that this confusion centers on the metric rather than the methodology. Calculating a decision curve requires only the most trivial math [1], but the two axes—threshold probability and net benefit—are concepts that are novel to many.

We hope that this didactic overview will aid in the interpretation of decision curve analysis and ensure that the basic concepts underpinning decision curves are more widely understood.

## Data Availability

Not applicable

## Abbreviations

AUC: Area under the curve
*p̂*: Predicted probability that a given patient has the event of interest
PSA: Prostate-specific antigen
*p*_t_: Threshold probability
